# Correction: Handling and managing bleeding wounds using tissue adhesive hydrogel: a comparative assessment on two different hydrogels

**DOI:** 10.1039/c9ra90054c

**Published:** 2019-07-15

**Authors:** Thiruselvi T, Thirupathi Kumara Raja S, Aravindhan R, Shanuja S. K, Gnanamani A

**Affiliations:** CSIR-CLRI (Central Leather Research Institute) Adyar Chennai-20 Tamil Nadu India gnanamani3@gmail.com +91-44-24912150

## Abstract

Correction for ‘Handling and managing bleeding wounds using tissue adhesive hydrogel: a comparative assessment on two different hydrogels’ by Thiruselvi T *et al.*, *RSC Adv.*, 2016, **6**, 19973–19981.

The authors regret that Fig. 3(a) in the original article included an incorrect image. The top right image (48 hours control) was duplicated as the middle right image (48 hours PEG-gel) in error. The correct version of Fig. 3 is presented here.

**Fig. 3:**
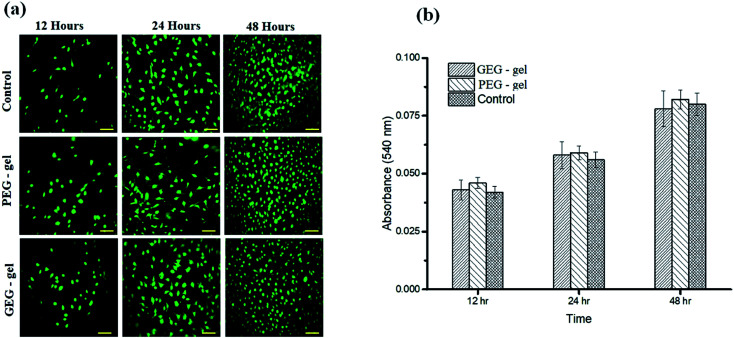
(a) Cytocompatibility assessments of the samples (PEG-gel and GEG-gel) through live cell tracker assay using calcein as a fluorescent probe demonstrates the cell adherence and proliferation observed at different time intervals in comparison with control (uncoated wells). The scale bar measures 10 μm. (b) Cytotoxicity assessment of the samples (PEG-gel and GEG-gel) using MTT assay by measuring the absorbance at 570 nm in comparison with control.

The Royal Society of Chemistry apologises for these errors and any consequent inconvenience to authors and readers.

## Supplementary Material

